# Taking the Next Step in Personalised Orthopaedic Implantation

**DOI:** 10.3390/jpm12030365

**Published:** 2022-02-27

**Authors:** Maximilian Rudert

**Affiliations:** Orthopaedic Department, König-Ludwig-Haus, University of Wuerzburg, D-97074 Wuerzburg, Germany; m-rudert.klh@uni-wuerzburg.de

Most of the treatments in medicine are patient specific, are they not?

We examine patients, make individual diagnoses and adapt our therapy to the specific case. The more precisely we are in our efforts to record all parameters that could influence our therapy, the more individual our treatment will become for the patient. Machine Learning, Neural Networks, and Big Data management will help us to overcome the endless story of biomedical statistical approaches concerning outcome data in orthopaedics [1]. Hopefully we can find out in the long term who exactly is suitable for which therapy. One problem in orthopaedics is that new treatment approaches, such as patient-specific instruments, are applied equally to all patients without it being possible to demonstrate differences in the treatment result.

Why should we bother with individualization of our implants and techniques, if we adapt our therapy to patients anyway? The more we try to pigeonhole the patient, which is often forced to us by treatment guidelines and classifications we use, the more likely we are not to achieve individual treatment. Looking at the neighbouring field of oncologic treatment, nobody would question that individualization of tumour therapy with personalized instruments like antibodies has led to thriving of this field in terms of success in patient survival and positive responses to alternatives for conventional treatments.

The same seems to happen to the field of orthopaedic surgery although not strikingly obvious because outcome does not equal survival in most of our cases.

Nonetheless, tumour surgery is a good way of looking at things in orthopaedic personalization, but from a different angle. The defects that arise from tumours and their surgical removal are so different that only in rare cases do they not require any adjustment to the standard [2]. This has been the case for decades, but the techniques that are available to us are becoming more and more sophisticated and allow better restorations [3,4,5,6]. The same is actually true for defects in revision arthroplasty and spinal surgery [7,8,9]. Since more and more revisions are re-revisions, the defects and collateral damage become bigger with every episode of loosening and consecutive operation. 3D printing technologies allow for visualization of the defects on templates that can be used to understand our treatment approach. Still, in order to simplify the treatment, we classify the defects, which, firstly, is not easy and, secondly, does not always make sense [10,11]. Again we come into the dilemma of small numbers, very individual anatomical requirements and a multitude of confounders, which make a statistical analysis practically impossible [1]. Furthermore, three-dimensional defects can change during the operative procedure. A lot of experience is therefore necessary in order to anticipate this and either fit bones using resection guides or tolerate inaccuracies where possible.

Modern alignment techniques in primary knee arthroplasty have also adopted the concepts of individualization of the implant position and soft tissue tension to approximate the preoperative situation. In this way, the individual anatomical requirements of the patient are taken into account and the joint is not put into a predicament in which it has never been before. This is intended to increase the outcome and satisfaction [12,13,14]. The use of patient-specific implants in primary endoprosthetics follows a comparable principle. Due to the optimal alignment and size as well as the shape of the implants, the physiological load transfer should be maintained in the entire movement of the joints [15,16,17,18]. 

Artificial intelligence-based recognition of different types of implants and detection of the region of interest in standard x-rays will help to improve treatment by gathering big data [19,20]. With these large amounts of data, it will be easier to optimize standard situations for the individual patient and his or her specific anatomical requirements [21,22]. Ultimately, however, this information only helps us if we can put it into practice on the patient. Augmented reality and robotic systems will help us to translate the planning [23]. Personally, I strongly believe that these new technologies will bring us further in successful and, above all, adapted therapy for our patients.

## Data Availability

See References to the Editorial.
